# Malaria control adds to the evidence for health aid effectiveness

**DOI:** 10.1371/journal.pmed.1002320

**Published:** 2017-06-13

**Authors:** Eran Bendavid

**Affiliations:** Stanford University, Stanford, California, United States of America

## Abstract

In this Perspective, Eran Bendavid discusses the broader political and health consequences of US foreign aid in light of the study by Aleksandra Jakubowski and colleagues of the President's Malaria Initiative in sub-Saharan Africa.

The new United States administration’s first budget proposal, previewed in March and released in May, 2017, includes deep cuts to foreign aid, cycling this thorny issue back into the American limelight [1]. The stated reasons for the cuts are that “the United States currently pays more than its fair share,” and to ensure that “foreign aid supports American interests and values.” The budget proposal is not specific on which types of development assistance will undergo the largest budget cuts but, at 20% of all US foreign aid, the health sector may undergo substantial changes if the budget is implemented [2].

The implication in the President’s statement is that foreign aid does not align with the interests of—and may provide low value for—the American people. Views of foreign aid shift with popular perceptions about the roles of and benefits to the US from investing in the growth and development of other nations. Critics of foreign aid often portray it as a doubly wasteful endeavor: it doesn’t achieve its intended goals, and the resources could be spent productively on other priorities [3]. Images of corrupt officials exploiting the resources gifted or loaned to build bridges or dams that later crumble can be powerful in swaying attitudes towards foreign aid [4].

While the evidence and anecdotes questioning foreign aid have some validity when it comes to economic development, the story with health is very different [5]. In fact, US foreign aid for health has arguably been the single most important driver of the last 20 years’ health improvements in developing countries. No single country—rich or poor—has provided more financial support for expanding childhood vaccinations (both from US national sources and the Gates Foundation), for providing antiretroviral therapy at the height of the HIV epidemic, and for reinvigorating global efforts to fight malaria [6]. Perhaps the greatest crowning achievements of global health in the past generation, the halving of child mortality since 2000 and the reversal of the HIV-driven downward spiral of life expectancy across southern and eastern Africa, can be traced back to US-financed organizations [7, 8].

The US-financed retreat of malaria now adds to the pantheon of global health achievements. In this issue of *PLOS Medicine*, Aleksandra Jakubowski and colleagues provide an independent evaluation of the US President’s Malaria Initiative that adds meaningful evidence to the literature on health aid effectiveness [9]. The authors use the Initiative’s unique structure—it was implemented in only 19 sub-Saharan African countries—to carefully examine the historical record on where and when malaria interventions have been implemented. The authors look at the malaria technologies that the Initiative financed directly—insecticide-treated nets, artemisinin-based combination therapy, and indoor residual spraying—before examining under-five mortality. For all three technologies, the authors find an increase in coverage after the Initiative’s implementation that was greater in the countries where it was implemented compared with neighboring sub-Saharan African countries (the change was positive but not statistically significant for artemisinin-based combination therapy). The authors find that these increases in the coverage of efficacious malaria interventions were accompanied by an annual risk of under-five death that declined 15% more in the Initiative’s partner countries compared with neighboring countries following the Initiative’s implementation. These are striking findings.

Are these findings plausible in the context of evidence on aid effectiveness? One of the vexing dilemmas of foreign aid is that the “hydraulic” model (more in, more out) often fails empirical scrutiny, even when it is intended for efficacious interventions [5]. Efficacy (having a benefit in a trial setting) is commonly different from effectiveness (having a benefit in real-world context), and the efficacy–effectiveness gap is the bane of many working in global health and development [10]. Three features distinguish effective health aid: it (i) supports large programs (ii) that finance highly efficacious interventions (iii) in populations with high-burden and low-met need. This was the landscape for malaria when the US President’s Malaria Initiative started. After frequently successful efforts to control the *Anopheles* vector in the 1950s and 1960s, often using DDT, malaria control programs retreated and the epidemic took hold across most of sub-Saharan Africa [11]. National malaria control programs were chronically underfunded, especially in high-burden regions. The malaria mortality curve bent downward only after the resurgence of attention to the epidemiology, the development of low-cost insecticide-treated nets and artemisinin-based therapies, and the commitment by large foreign aid organizations.

The motto of the US Agency for International Development, pasted on every USAID-financed clinic, is “From the American People.” The American people do indeed fund USAID—about 1% of US government revenue goes to foreign aid. But the American people also benefit from the successes of foreign aid for health. Averting deaths of young children from malaria or vaccine-preventable diseases such as polio or measles promotes more stable and prosperous societies. In countries where the US gives most for health, the perception of the United States is among the most favorable in the world. For example, results from Pew Research Center Global Attitudes & Trends surveys show that in Ghana and Kenya, two partner countries for malaria and other forms of health aid, the portion of the population that views the US favorably approaches 90%, higher than in any European country (higher even than in the US) [12]. Finally, the economic benefits from reducing the burden of malaria are substantial, and some of those benefits are likely to return to the US [13].

The evidence in the study by Jakubowski and colleagues is the first demonstration of the large-scale effectiveness of foreign aid for malaria control, and it joins the canon supporting health aid effectiveness. It also underscores that the anticipated benefits of effective aid include not only a reduction in the number of children dying in poor countries, but also, arguably, an investment in the well-being of Americans.
